# A new prognostic histopathologic classification of nasopharyngeal carcinoma

**DOI:** 10.1186/s40880-016-0103-5

**Published:** 2016-05-05

**Authors:** Hai-Yun Wang, Yih-Leong Chang, Ka-Fai To, Jacqueline S. G. Hwang, Hai-Qiang Mai, Yan-Fen Feng, Ellen T. Chang, Chen-Ping Wang, Michael Koon Ming Kam, Shie-Lee Cheah, Ming Lee, Li Gao, Hui-Zhong Zhang, Jie-Hua He, Hao Jiang, Pei-Qing Ma, Xiao-Dong Zhu, Liang Zeng, Chun-Yan Chen, Gang Chen, Ma-Yan Huang, Sha Fu, Qiong Shao, An-Jia Han, Hai-Gang Li, Chun-Kui Shao, Pei-Yu Huang, Chao-Nan Qian, Tai-Xiang Lu, Jin-Tian Li, Weimin Ye, Ingemar Ernberg, Ho Keung Ng, Joseph T. S. Wee, Yi-Xin Zeng, Hans-Olov Adami, Anthony T. C. Chan, Jian-Yong Shao

**Affiliations:** State Key Laboratory of Oncology in South China; Collaborative Innovation Center for Cancer Medicine, Sun Yat-sen University Cancer Center, #651 Dongfeng Road East, Guangzhou, 510060 Guangdong P. R. China; Department of Molecular Diagnostics, Sun Yat-sen University Cancer Center, Guangzhou, 510060 Guangdong P. R. China; Department of Pathology, National Taiwan University Hospital, National Taiwan University College of Medicine, Taibei, 10002 Taiwan P. R. China; Department of Anatomical and Cellular Pathology, Hong Kong Cancer Institute and Sir YK Pao Centre for Cancer, Faculty of Medicine, The Chinese University of Hong Kong, Hong Kong, 10871 P. R. China; Department of Pathology, Singapore General Hospital, Singapore, 169608 Singapore; Department of Nasopharyngeal Carcinoma, Sun Yat-sen University Cancer Center, Guangzhou, 510060 Guangdong P. R. China; Department of Pathology, Sun Yat-sen University Cancer Center, Guangzhou, 510060 Guangdong P. R. China; Division of Epidemiology, Department of Health Research and Policy, Stanford University School of Medicine, Stanford, CA 94305 USA; Health Sciences Practice, Exponent, Inc., Menlo Park, CA 94025 USA; Department of Otolaryngology, National Taiwan University Hospital, National Taiwan University College of Medicine, Taibei, 10002 Taiwan P. R. China; Department of Clinical Oncology, Hong Kong Cancer Institute and Sir YK Pao Centre for Cancer, Faculty of Medicine, The Chinese University of Hong Kong, Hong Kong, 10871 P. R. China; Division of Radiation Oncology, National Cancer Center, Singapore, 169610 Singapore; Department of Radiation Oncology, Cancer Institute and Hospital, Chinese Academy of Medical Sciences, Beijing, 100730 P. R. China; Department of Radiation Oncology, The Affiliated Hospital of Bengbu Medical College, Bengbu, 233030 Anhui P. R. China; Department of Pathology, Cancer Institute and Hospital, Chinese Academy of Medical Sciences, Beijing, 100730 P. R. China; Department of Radiation Oncology, Guangxi Medical University, Nanning, 530021 Guangxi P. R. China; Department of Pathology, Hunan Provincial Cancer Hospital, Changsha, 410013 Hunan P. R. China; Department of Radiation Oncology, Sun Yat-sen University Cancer Center, Guangzhou, 510060 Guangdong P. R. China; Department of Pathology, Fujian Provincial Cancer Hospital, Fuzhou, 350014 Fujian P. R. China; Department of Pathology, The First Affiliated Hospital, Sun Yat-sen University, Guangzhou, 510080 Guangdong P. R. China; Department of Pathology, Sun Yat-sen Memorial Hospital, Sun Yat-sen University, Guangzhou, 510120 Guangdong P. R. China; Department of Pathology, The Third Affiliated Hospital, Sun Yat-sen University, Guangzhou, 510630 Guangdong P. R. China; Department of Experiment Research, Sun Yat-sen University Cancer Center, Guangzhou, 510060 Guangdong P. R. China; Department of Medical Epidemiology and Biostatistics, Karolinska Institutet, Stockholm, 171 77 Sweden; Departments of Microbiology and Tumor Biology Center, Karolinska Institutet, Stockholm, 171 77 Sweden; Department of Epidemiology, Harvard School of Public Health, Boston, MA 02115 USA

**Keywords:** Nasopharyngeal carcinoma, Pathologic classification, Prognosis

## Abstract

**Background:**

The current World Health Organization (WHO) classification of nasopharyngeal carcinoma (NPC) conveys little prognostic information. This study aimed to propose an NPC histopathologic classification that can potentially be used to predict prognosis and treatment response.

**Methods:**

We initially developed a histopathologic classification based on the morphologic traits and cell differentiation of tumors of 2716 NPC patients who were identified at Sun Yat-sen University Cancer Center (SYSUCC) (training cohort). Then, the proposed classification was applied to 1702 patients (retrospective validation cohort) from hospitals outside SYSUCC and 1613 patients (prospective validation cohort) from SYSUCC. The efficacy of radiochemotherapy and radiotherapy modalities was compared between the proposed subtypes. We used Cox proportional hazards models to estimate hazard ratios (HRs) with 95% confidence intervals (CI) for overall survival (OS).

**Results:**

The 5-year OS rates for all NPC patients who were diagnosed with epithelial carcinoma (EC; 3708 patients), mixed sarcomatoid-epithelial carcinoma (MSEC; 1247 patients), sarcomatoid carcinoma (SC; 823 patients), and squamous cell carcinoma (SCC; 253 patients) were 79.4%, 70.5%, 59.6%, and 42.6%, respectively (*P* < 0.001). In multivariate models, patients with MSEC had a shorter OS than patients with EC (HR = 1.44, 95% CI = 1.27–1.62), SC (HR = 2.00, 95% CI = 1.76–2.28), or SCC (HR = 4.23, 95% CI = 3.34–5.38). Radiochemotherapy significantly improved survival compared with radiotherapy alone for patients with EC (HR = 0.67, 95% CI = 0.56–0.80), MSEC (HR = 0.58, 95% CI = 0.49–0.75), and possibly for those with SCC (HR = 0.63; 95% CI = 0.40–0.98), but not for patients with SC (HR = 0.97, 95% CI = 0.74–1.28).

**Conclusions:**

The proposed classification offers more information for the prediction of NPC prognosis compared with the WHO classification and might be a valuable tool to guide treatment decisions for subtypes that are associated with a poor prognosis.

## Background

Nasopharyngeal carcinoma (NPC) is endemic in North Africa and Southeast Asia and most notably in South China, where the incidence can be as high as 20–40 per 100,000 persons [1–3]. NPC differs from other head and neck cancers with regard to epidemiologic features, histopathologic features, treatment strategies, and response to therapy [4, 5]. Based on the current World Health Organization (WHO) pathologic classification, NPCs are grouped into keratinizing squamous cell carcinoma (KSCC) and non-keratinizing carcinoma. The latter group is further subdivided into non-keratinizing differentiated carcinoma (NKDC) and non-keratinizing undifferentiated carcinoma (NKUC). However, this system is insufficiently informative, as clinical outcomes vary substantially among patients with the same clinical stage and histopathologic subtype [6–8]. Prognosis does not differ significantly between the NKUC and NKDC subtypes [8–10]. Pathologists have observed that NPC tumor cells have obvious morphologic variations, with cells that are small and round, large and round, spindle-shaped, with or without vesicular nuclei, or mixed round and spindle-shaped. Despite this morphologic heterogeneity, proposed NPC histopathologic classifications to date have not demonstrated clinically relevant improvement in prognostic prediction beyond the WHO classification [11]. Therefore, clinicians have continued to appeal to pathologists to propose an NPC histopathologic classification system that better predicts prognosis and that enables personalized treatment of NPC patients.

In advanced NPC, radiochemotherapy (RCT) has been extensively investigated and demonstrated to improve tumor control and patient survival [12–17]. Two recent trials reported no survival benefit of concurrent RCT plus adjuvant chemotherapy versus concurrent RCT for advanced NPC; in addition, no survival benefit was reported with induction chemotherapy plus concurrent RCT versus induction chemotherapy plus radiotherapy (RT) alone for advanced NPC [16, 18]. Therefore, more accurate prognostication is needed to avoid over-treatment and to tailor treatment strategies that are compatible with individual risk patterns in a manner that improves patients’ survival outcomes.

The primary objective of this large, multi-center study was to propose a histopathologic classification system for NPC that offers more information on prognosis than the WHO classification. A secondary, more exploratory objective was to determine whether patients with each histopathologic subtype benefit equally in terms of overall survival (OS) after RCT versus RT alone.

## Methods

### Study design

**Fig. 1:**
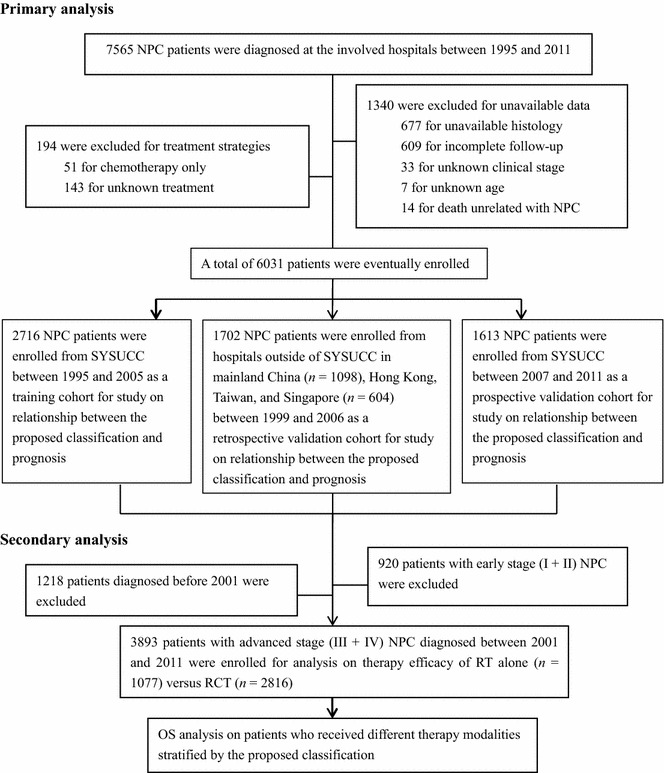
Flowchart of the study design shows the inclusion and exclusions of nasopharyngeal carcinoma (NPC) patients with different therapeutic modalities stratified by the proposed classification. *SYSUCC* Sun Yat-sen University Cancer Center, *OS* overall survival, *RT* radiotherapy, *RCT* radiochemotherapy

Figure 1 describes the criteria of patient selection and exclusion. In detail, the inclusion criteria were as follows: the availability of hematoxylin and eosin (H&E) slides for review, the availability of follow-up data, no history of other treated cancer, and appropriate informed consent from patients. Patients with unknown treatment, unknown age or clinical stage, and those who received chemotherapy alone were excluded from this study. Patients who died from causes unrelated to NPC were also excluded. Two clinical staging systems were applied due to different geographic areas: patients from mainland China enrolled before 2006 were staged according to the 1992 China staging system [19], whereas patients from Hong Kong, Taiwan, Singapore, and Sun Yat-sen University Cancer Center (SYSUCC) who were enrolled between 2007 and 2011 were staged according to the 1997 American Joint Committee on Cancer (AJCC) staging system [20]. The ethics committee or institutional review board at each participating center approved the study.

### Morphologic features of each subtype of the proposed classification of NPC

**Fig. 2:**
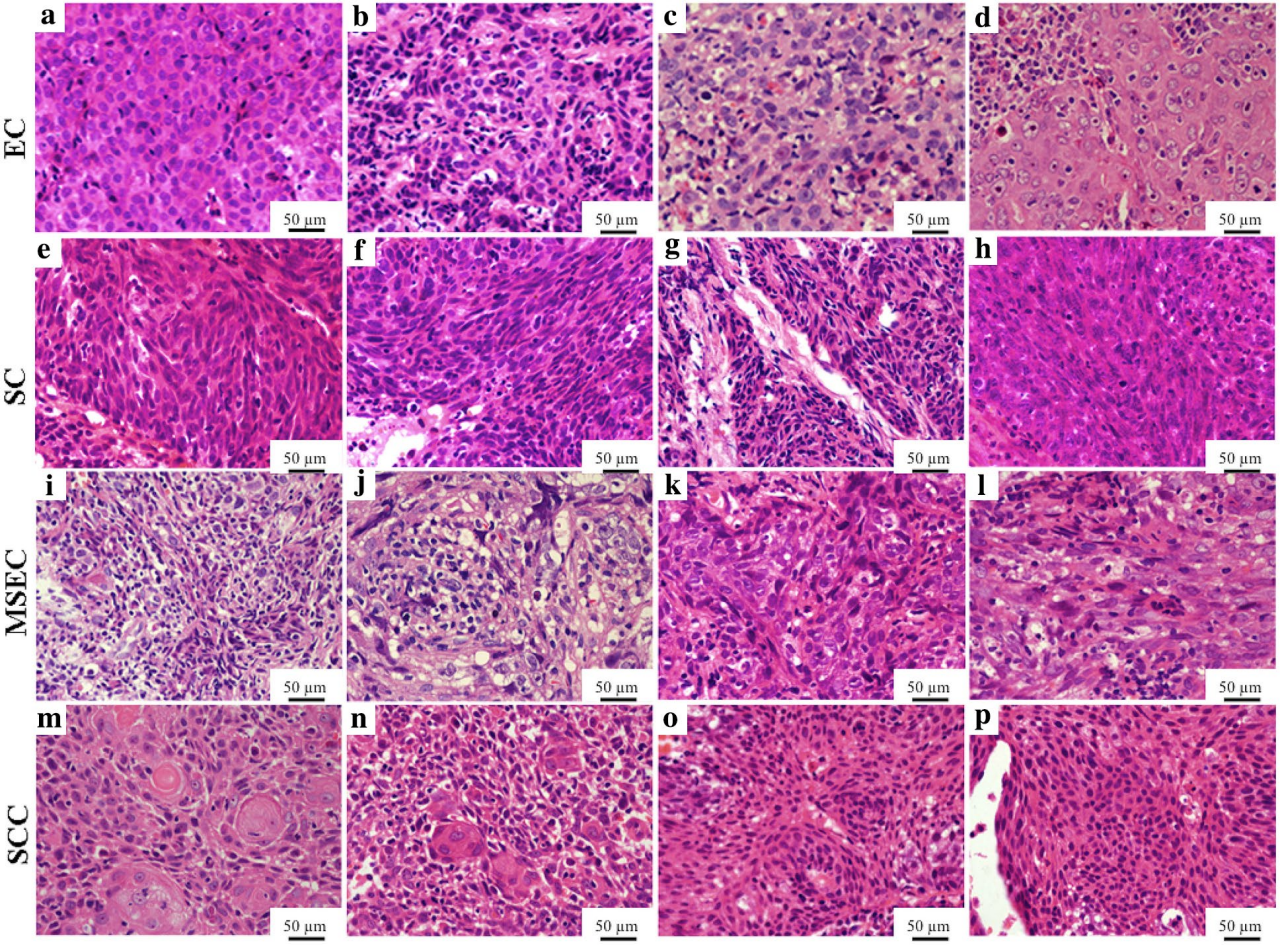
Representative morphologic traits of tumors according to the proposed classification of NPC (H&E, ×400). The epithelial carcinoma (EC) subtype shows small, *round cells* with cellular stratification and a pavement-like appearance, a low nucleus:cytoplasm ratio, chromatin-rich nuclei (**a**), and non-prominent nucleoli (**b**); or syncytial-appearing large tumor cells with indistinct cell borders, round-to-oval vesicular nuclei, and large central nucleoli (**c**); a round shape with vesicular nuclei and prominent nucleoli accounting for more than 75% of tumor cells (**d**). The sarcomatoid carcinoma (SC) subtype features irregular small cells, large hyperchromatic cells, or both, or uniformly medium-sized spindle cells (**e**), together with nucleoli that are less prominent than those in the syncytial-appearing cells (**f**), dark, smudged nuclei and a dense amphophilic (**g**), or eosinophilic cytoplasm (**h**). The mixed sarcomatoid-epithelial carcinoma (MSEC) subtype is characterized by large, round cell nests (**i**) or scattered infiltration of large, round cells in the spindle cell carcinomatous tissue (**j**); no obvious boundaries were observed between the tumor and interstitial lymphoid tissue (**k**) or in the stromal portion that contained cells with eosinophilic cytoplasm (**l**). The squamous cell carcinoma (SCC) subtype shows well differentiated keratinizing SCC with a large number of whorls (**m**) and keratin (**n**), or poorly or moderately differentiated SCC with some individual keratinized spine cells (**o**) and a small number of basal-like cells (**p**)

Representative features of each subtype of the proposed classification are shown in Fig. 2. NPCs were histologically classified into the following four subtypes based on morphologic features: epithelial carcinoma (EC), sarcomatoid carcinoma (SC), mixed sarcomatoid-epithelial carcinoma (MSEC), and squamous cell carcinoma (SCC). Specifically, EC is characterized by small, round tumor cells (Fig. 2a, b), large, round cells (Fig. 2c), or a carcinoma phenotype with vesicular nuclei (Fig. 2d). More than 50% of the tumor cells in SC are spindle-shaped, fusiform, or are in interlacing bundles (fibrosarcomatous pattern) (Fig. 2e–h). Morphologically, MSEC shows nests or scattered infiltration of large, round cells in spindle cell carcinomatous tissues (Fig. 2i–l). SCC is distinguished by tumor cells with a well differentiated keratinizing phenotype (Fig. 2m, n) or a poorly or moderately differentiated phenotype (Fig. 2o, p).

### Inter-observer reproducibility of slide review

Two experienced pathologists without knowledge of the clinical data independently classified all enrolled cases from each participating institution according to the proposed classification, and simultaneously reclassified all cases according to the WHO classification criteria. A third pathologist from the institution was consulted when the classifications of the first two pathologists conflicted. If the conclusion of the third pathologist was different, then the three worked collaboratively to reach an agreement. Inter-observer reproducibility of the results between the first two pathologists according to the new classification was 90.2% (Table 1).

**Table 1:** Inter-observer reproducibility between two pathologists based on the new nasopharyngeal carcinoma (NPC) histopathologic classification, stratified by area of case origin

Case origin	Consistent (cases)	Inconsistent (cases)	Reproducibility (%)
Sun Yat-sen University Cancer Center (SYSUCC)	3067	325	90.3
Other hospitals in mainland China	582	53	91.7
Hong Kong, Taiwan, Singapore	535	77	87.4
Total	4187	455	90.2

In all, 4642 out of the 7565 patients in the initial database were assessed for the initial calculation of reproducibility

### Efficacy of radiotherapy and radiochemotherapy

The patients with advanced NPC who were diagnosed between 2001 and 2011 underwent further analysis of the therapeutic efficacy of RCT versus RT alone and were then stratified by the proposed classification; the patients who were diagnosed before January 1, 2001, and those with early-stage disease were excluded to reduce variation in treatment protocols (Fig. 1). All patients with advanced NPC underwent standard curative RT, and some received additional chemotherapy. Patients in both groups received RT according to the policy at each center. The treatment protocol used at the largest contributing center was reported previously [21]. Generally, a regimen that consisted of 2 Gy per fraction, with 5 daily fractions per week, was used. A minimum dose of 60 Gy was given to gross tumor targets, while 50 Gy was given at sites of local infiltration and bilateral cervical lymphatic metastases. Patients who received RCT were administered neoadjuvant, concurrent, or adjuvant cisplatin (30–40 mg/m^2^ every week or 100 mg/m^2^ every 3 weeks) plus 5-fluorouracil (750 mg/m^2^ per day, days 1–5).

### Statistical analysis

Probabilities of OS were estimated using the Kaplan–Meier method, and the log-rank test was used to detect differences among groups. We assessed the associations between clinical characteristics and subtypes of NPC classified according to the proposed classification using Student’s *t* test and the Chi square test. To test if the proposed classification was an independent prognostic factor of OS, we adjusted for age, sex, clinical stage, therapeutic modality, and the WHO classification and used multivariate Cox proportional hazards regression models to estimate hazard ratios (HRs) with 95% confidence intervals (CI). The heterogeneity according to clinical stage in the association between histopathologic classification and OS was examined by the inclusion of an interaction term between stage and histology and by the stratification of models by stage at diagnosis. The patients were followed every 6 months, and a 5-year follow-up was achieved for 75.9% (4583/6031) of the NPC patients. However, enrollment in the prospective validation cohort continued until as recently as 2011, and follow-up data were recorded until April 2, 2015; therefore, the 5-year follow-up rate was 38.5% in this cohort. We calculated the OS from the date of diagnosis until the date of death of NPC or the last date of follow-up. All statistical tests were two-sided, and a *P* value of less than 0.05 was considered statistically significant. All statistical analyses were performed with Stata software (version 13, the University of California, Los Angeles, CA, USA).

## Results

### Patient characteristics

This study enrolled 6031 patients with newly diagnosed, histologically confirmed, previously untreated NPC: 4329 were enrolled from SYSUCC in Guangzhou, China, 604 from Hong Kong, Taiwan, and Singapore, and 1098 from other institutions in mainland China (Table 2). Table 3 summarizes the demographic and clinical characteristics of NPC patients in the training, retrospective validation, and prospective validation cohorts, who were followed up for a median of 68, 68, and 41 months, respectively. NPC patients in the training cohort (*n* = 2716) from SYSUCC and the retrospective validation cohort from other hospitals in mainland China (*n* = 1098) were staged according to the 1992 China staging system. Patients from Hong Kong, Taiwan, and Singapore in the retrospective validation cohort (*n* = 604) as well as patients from SYSUCC in the prospective validation cohort (*n* = 1613) were staged according to the 1997 AJCC staging system (Fig. 1).

**Table 2:** Distribution of clinical centers where the 6031 enrolled nasopharyngeal carcinoma patients received a biopsy and therapy

Center for biopsy	Center for therapy	Number of cases
Department of Pathology, Sun Yat-sen University Cancer Center (SYSUCC), Guangzhou, Guangdong, China	Department of Radiation Oncology, SYSUCC	4329
Department of Pathology, multiple local centers in Guangdong, China	Department of Radiation Oncology, SYSUCC	504
Department of Pathology, Guangxi Medical University Cancer Center, Nanning, Guangxi, China	Department of Radiation Oncology, Guangxi Medical University Cancer Center	204
Department of Pathology, Cancer Institute and Hospital, Chinese Academy of Medical Sciences (CAMS), Beijing, China	Department of Radiation Oncology, Cancer Hospital, CAMS	100
Department of Pathology, The Affiliated Hospital of Bengbu Medical College, Bengbu, Anhui, China	Department of Radiation Oncology, The Affiliated Hospital of Bengbu Medical College	113
Department of Pathology, Hunan Provincial Cancer Hospital, Changsha, Hunan, China	Department of Radiation Oncology, Hunan Provincial Cancer Hospital	130
Department of Pathology, Fujian Provincial Tumor Hospital, Fuzhou, Fujian, China	Department of Radiation Oncology, Fujian Provincial Tumor Hospital	47
Department of Anatomical and Cellular Pathology, Faculty of Medicine, the Chinese University of Hong Kong (CUHK), Hong Kong, China	Department of Clinical Oncology, CUHK	207
Department of Pathology, National Taiwan University Hospital, Taipei, Taiwan	Department of Otolaryngology, National Taiwan University Hospital	211
Department of Pathology, Singapore General Hospital, Singapore, Singapore	Department of Radiation Oncology, National Cancer Center, Singapore	186

**Table 3:** Clinical characteristics of NPC patients in the training, retrospective validation, and prospective validation cohorts

Characteristic	Training cohort	Retrospective validation cohort	Prospective validation cohort
Total (cases)	2716	1702	1613
Age (years)			
Median (range)	46 (10–86)	47 (10–90)	47 (11–83)
Follow-up time (months)			
Median (range)	68 (1–120)	68 (1–120)	41 (1–91)
Sex [cases (%)]			
Female	658 (24.2)	501 (29.4)	404 (25.1)
Male	2058 (75.8)	1201 (70.6)	1209 (74.9)
Clinical stage [cases (%)]			
I	75 (2.8)	67 (4.0)	35 (2.2)
II	549 (20.2)	388 (22.8)	104 (6.5)
III	1272 (46.8)	695 (40.8)	458 (28.4)
IV	820 (30.2)	552 (32.4)	1016 (62.9)
Therapeutic modality [cases (%)]			
Radiotherapy alone	1592 (58.6)	928 (54.5)	220 (13.6)
Radiochemotherapy	1124 (41.4)	774 (45.5)	1393 (86.4)
Proposed classification [cases (%)]			
EC	1520 (55.9)	987 (58.0)	1202 (74.5)
MSEC	598 (22.0)	420 (24.7)	229 (14.2)
SC	489 (18.0)	198 (11.6)	135 (8.4)
SCC	109 (4.1)	97 (5.7)	47 (2.9)
WHO classification [case (%)]			
NKUC	2261 (83.2)	1425 (83.8)	1185 (73.5)
NKDC	409 (15.1)	227 (13.3)	414 (25.7)
KSCC	46 (1.7)	50 (2.9)	14 (0.8)
OS rate (%)			
5-year (95% CI)	68.7 (66.9–70.5)	73.3 (71.1–75.4)	83.5 (81.1–85.6)

*NPC* nasopharyngeal carcinoma, *EC* epithelial carcinoma, *SC* sarcomatoid carcinoma, *MSEC* mixed sarcomatoid-epithelial carcinoma, *SCC* squamous cell carcinoma, *WHO* World Health Organization, *NKUC* non-keratinizing undifferentiated carcinoma, *NKDC* non-keratinizing differentiated carcinoma, *KSCC* keratinizing squamous cell carcinoma, *OS* overall survival, *CI* confidence interval

### The proposed classification of NPC and patient survival

**Fig. 3:**
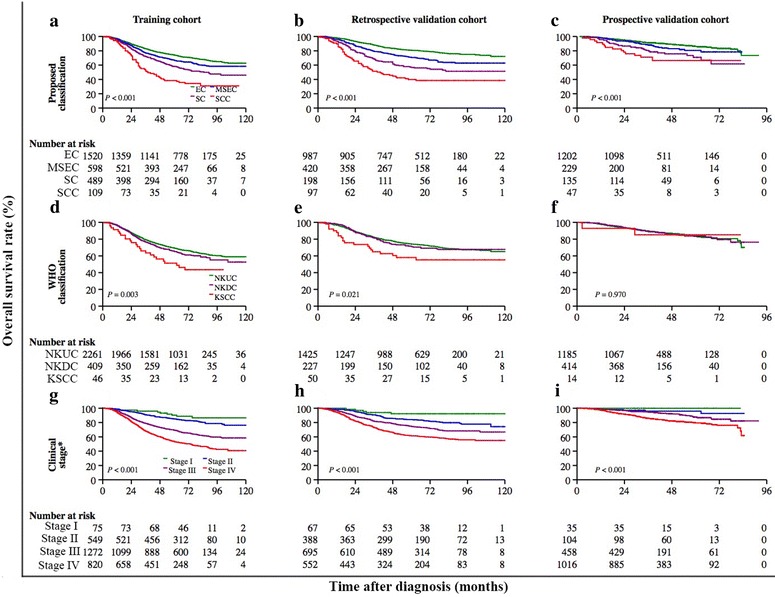
Kaplan-Meier curves of OS according to the proposed classification, the World Health Organization (WHO) classification, and clinical stage in NPC patients from the training (*n* = 2716), retrospective validation (*n* = 1702), and prospective validation cohorts (*n* = 1613), respectively. OS curves of patients classified with the proposed classification in the training (**a**), retrospective validation (**b**), and prospective validation cohorts (**c**). OS curves of patients classified with WHO classification in the training (**d**), retrospective validation (**e**), and prospective validation cohorts (**f**). OS curves of patients classified with clinical stage in the training (**g**), retrospective validation (**h**), and prospective validation cohorts (**i**). The log-rank test was used to estimate *P* values. *The training cohort was classified by the 1992 China staging system; the retrospective validation cohort was classified by the 1992 China staging system or the 1997 American Joint Committee on Cancer (AJCC) staging system; the prospective validation cohort was classified by the 1997 AJCC staging system

The 5-year OS rates in the training, retrospective validation, and prospective validation cohorts were 68.7%, 73.3%, and 83.5%, respectively (Table 3). Figure 3 displays that the differences in OS curves were significant for patients classified with the proposed classification system (*P* < 0.001) as well as for patients classified with clinical stage (*P* < 0.001) in each cohort. However, no difference was observed between the NKUC and NKDC subtypes, although the difference in OS of patients stratified by WHO classification remained significant in the training cohort (*P* = 0.003) and retrospective validation cohort (*P* = 0.021).

Because the association between the proposed classification and the 5-year OS was similar across the three cohorts, we combined all 6031 NPC patients for subsequent analysis. In all NPC patients, the 5-year OS rates for the EC, MSEC, SC, and SCC subtypes were 79.4%, 70.5%, 59.6%, and 42.6%, respectively (Table 4). The difference in the 5-year OS rate was 8.9% between the most common subtypes (EC and MSEC), which together comprised 82.2% of all patients; the difference was 19.8% between the EC and SC subtypes, which together comprised 75.1% of all patients; the difference was 17.0% between the poor-prognosis subtypes SC and SCC, which together comprised 17.8% of all patients in the study. These significant differences indicate that the proposed classification can distinguish the prognosis of NPC patients. By contrast, according to the WHO classification, a difference of only 0.6% was detected between the two most common subtypes (NKUC and NKDC), which together comprised 98.2% of all patients. NKUC cases were reclassified by the proposed classification primarily as EC (60.0%) and as MSEC (24.2%), whereas NKDC cases were reclassified primarily as EC (81.3%) (Chi square test, *P* < 0.001).

**Table 4:** Cox proportional regression analysis of the associations between patient characteristics and OS in all 6031 NPC patients

Characteristic	Patients [cases (%)]	OS rate (%)		Unadjusted HR	95% CI	*P* value	Adjusted HR^a^	95% CI	*P* value
		5-year	95% CI						
Proposed classification									
EC	3708 (61.5)	79.4	78.1–80.8	1.00	Reference		1.00	Reference	
MSEC	1247 (20.7)	70.5	67.7–73.0	1.48	1.32–1.67	<0.001	1.44	1.27–1.62	<0.001
SC	823 (13.6)	59.6	55.9–63.1	2.16	1.90–2.44	<0.001	2.00	1.76–2.28	<0.001
SCC	253 (4.2)	42.6	35.8–49.3	3.56	2.96–4.28	<0.001	4.23	3.34–5.38	<0.001
Age									
≤47 years	3163 (52.5)	77.8	76.3–79.3	1.00	Reference		1.00	Reference	
>47 years	2868 (47.5)	68.4	66.6–70.2	1.54	1.40–1.70	<0.001	1.50	1.36–1.65	<0.001
Sex									
Male	4468 (74.1)	71.7	70.3–73.1	1.00	Reference		1.00	Reference	
Female	1563 (25.9)	78.1	75.8–80.2	0.73	0.65–0.81	<0.001	0.76	0.68–0.86	<0.001
Therapeutic modality									
Radiotherapy alone	2739 (45.4)	71.4	69.6–73.1	1.00	Reference		1.00	Reference	
Radiochemotherapy	3292 (54.6)	75.2	73.4–76.8	0.88	0.79–0.97	0.009	0.65	0.59–0.72	<0.001
Clinical stage									
I	177 (2.9)	92.4	87.1–95.5	1.00	Reference		1.00	Reference	
II	1041 (17.3)	85.6	83.2–87.6	2.20	1.27–3.79	0.004	2.25	1.3–3.89	0.003
III	2424 (40.2)	74.3	72.4–76.0	4.17	2.46–7.08	<0.001	4.70	2.76–7.98	<0.001
IV	2389 (39.6)	64.9	62.7–67.0	5.77	3.40–9.78	<0.001	6.96	4.09–11.85	<0.001
WHO classification									
NKUC	4871 (80.8)	73.8	72.5–75.1	1.00	Reference		1.00	Reference	
NKDC	1050 (17.4)	73.2	70.1–75.9	1.02	0.90–1.16	0.729	1.00	0.87–1.16	0.980
KSCC	110 (1.8)	57.5	47.0–66.6	1.85	1.38–2.47	<0.001	0.51	0.35–0.74	<0.001

*OS* overall survival, *NPC* nasopharyngeal carcinoma, *HR* hazard ratio, *CI* confidence interval, *EC* epithelial carcinoma, *MSEC* mixed sarcomatoid-epithelial carcinoma, *SC* sarcomatoid carcinoma, *SCC* squamous cell carcinoma, *WHO* World Health Organization, *NKUC* non-keratinizing undifferentiated carcinoma, *NKDC* non-keratinizing differentiated carcinoma, *KSCC* keratinizing squamous cell carcinoma

^a^ All models were adjusted for the proposed classification, age, sex, therapeutic modality, clinical stage, and WHO classification

### The association between clinical stage and the proposed NPC classification

**Fig. 4:**
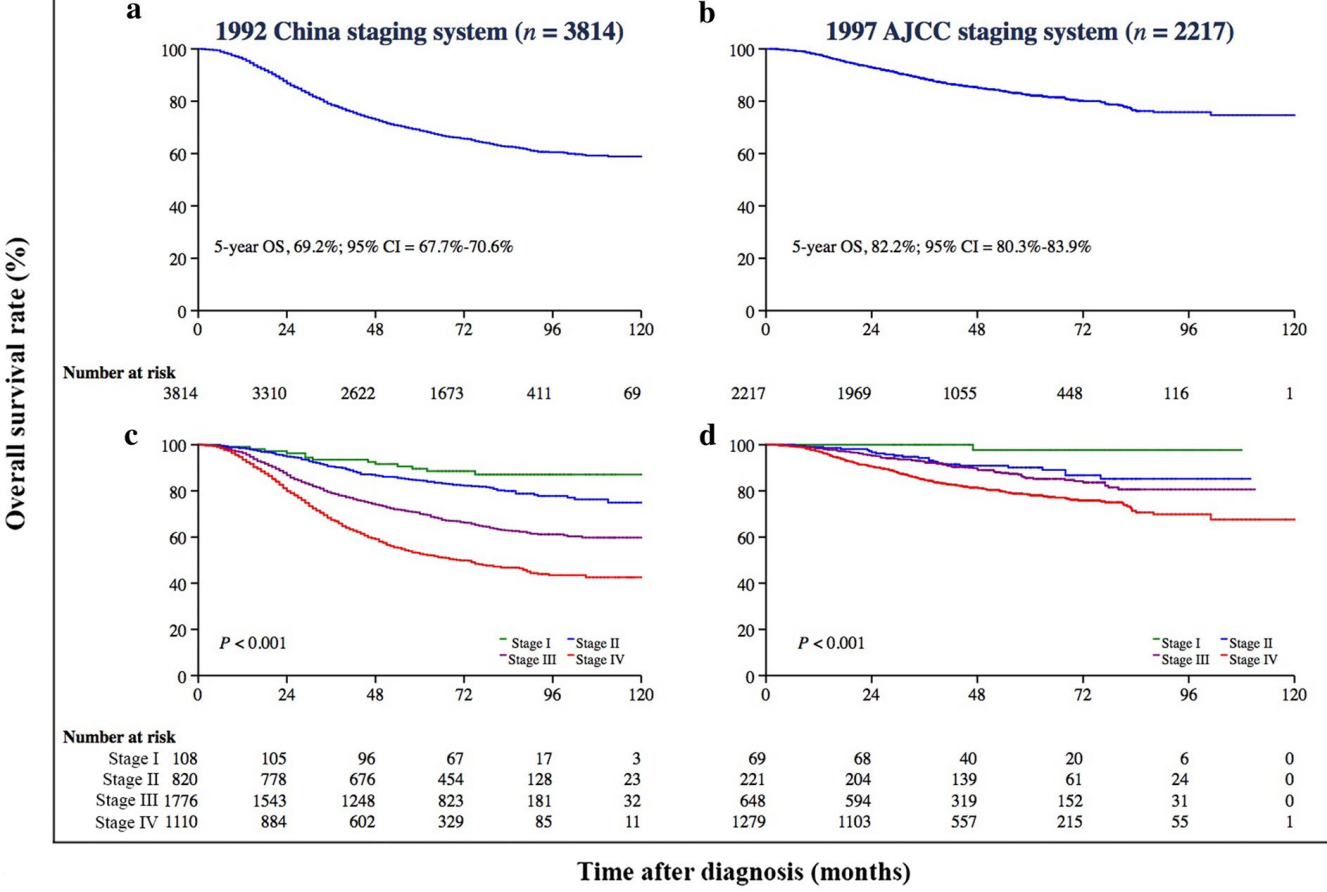
Kaplan-Meier survival estimates for NPC patients according to two different staging systems. In all, 3814 (63.2%) NPC patients from mainland China were diagnosed before 2006 and were staged according to the 1992 China staging system, whereas 2217 (36.8%) from Hong Kong, Taiwan, Singapore, and SYSUCC (in mainland China) were diagnosed between 2007 and 2011 and were staged according to the 1997 AJCC staging system. The 5-year OS rate was 69.2% (95% CI = 67.7%–70.6%) for patients classified by the 1992 China staging system (**a**) and 82.2% (95% CI = 80.3%–83.9%) for patients classified by the 1997 AJCC staging system (**b**). The OS differed significantly by clinical stage according to the 1992 China staging system (*P* < 0.001, **c**) and the 1997 AJCC staging system (*P* < 0.001, **d**). The log-rank test was used to calculate *P* values

**Fig. 5:**
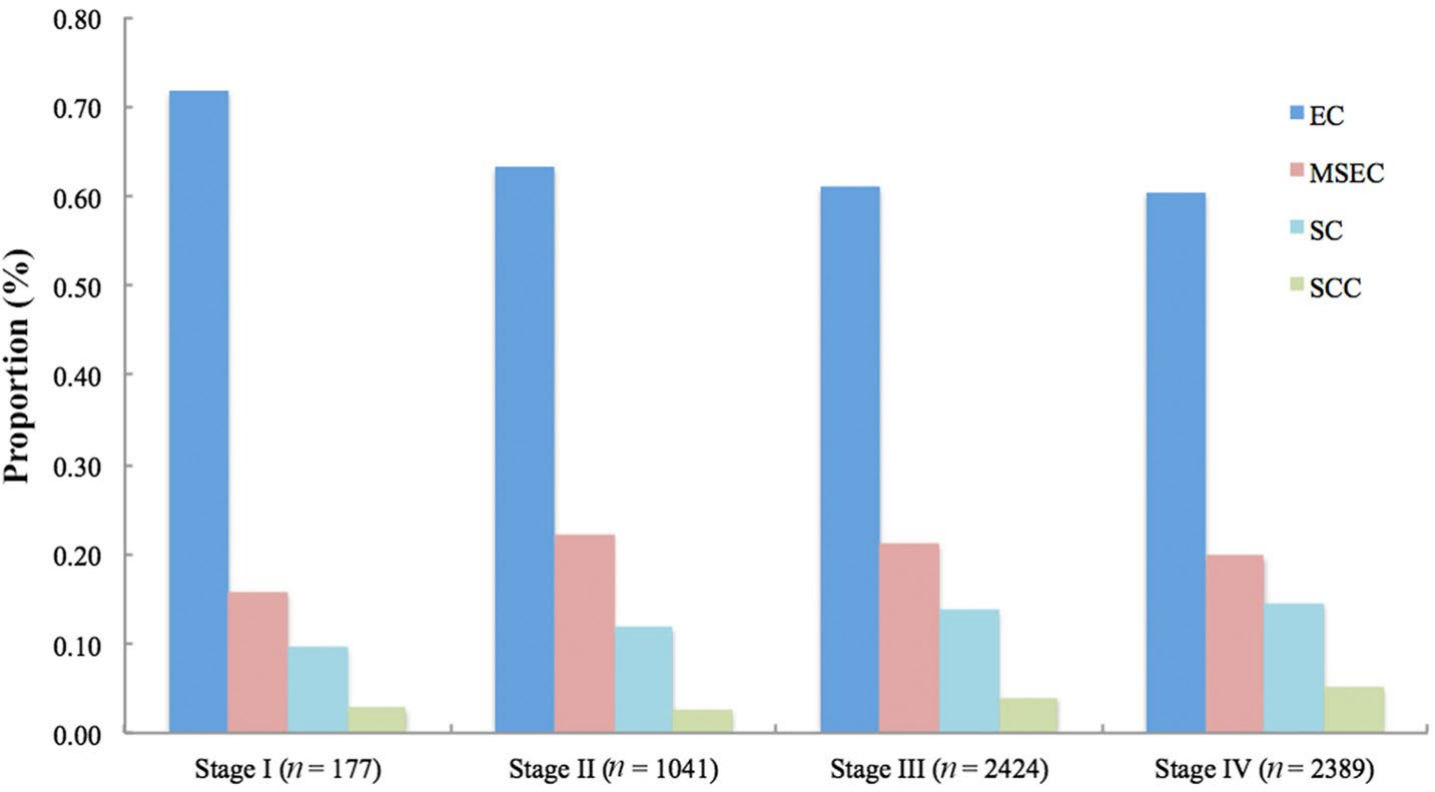
Associations between clinical stage and the proposed histopathologic classification in all NPC patients. The proportions of EC cases are 71.8%, 63.3%, 61.1%, and 60.4% in patients with stages I, II, III, and IV NPC (the 1992 China and 1997 AJCC staging systems combined), respectively, whereas those of SC cases are 9.6%, 11.8%, 13.7%, and 14.6%, respectively. The proportion of SC increased with advanced stage, whereas the proportion of EC decreased with more advanced stage (Chi square test, *P* = 0.001)

**Fig. 6:**
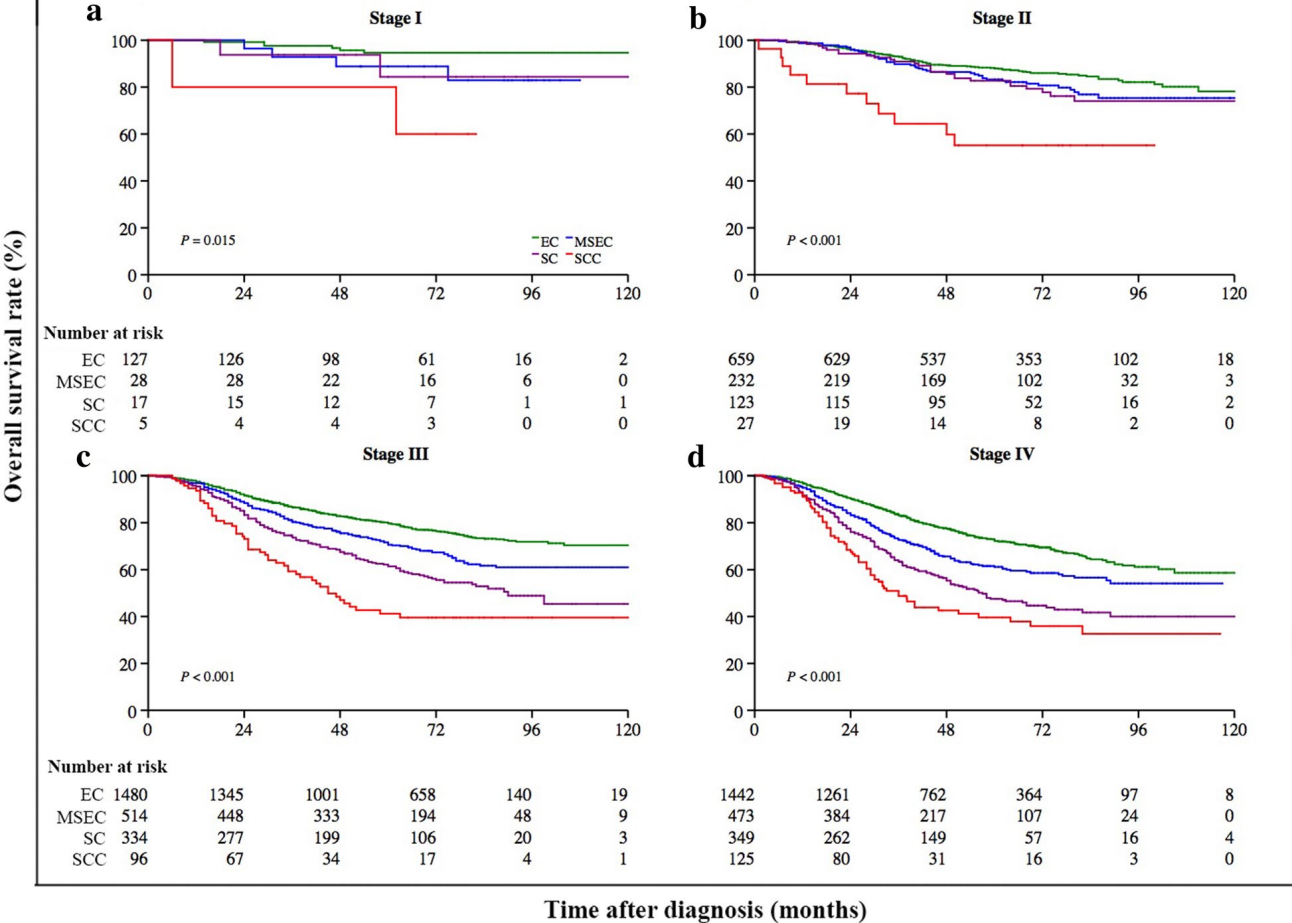
Kaplan-Meier survival estimates for NPC patients at different clinical stages (the 1992 China and 1997 AJCC staging systems combined) stratified by subtypes according to the proposed histopathologic classification. The OS differed significantly by the proposed classification for patients with stage I (*P* = 0.015, **a**), II (*P* < 0.001, **b**), III (*P* < 0.001, **c**), or IV disease (*P* < 0.001, **d**). The log-rank test was used to calculate *P* values

**Fig. 7:**
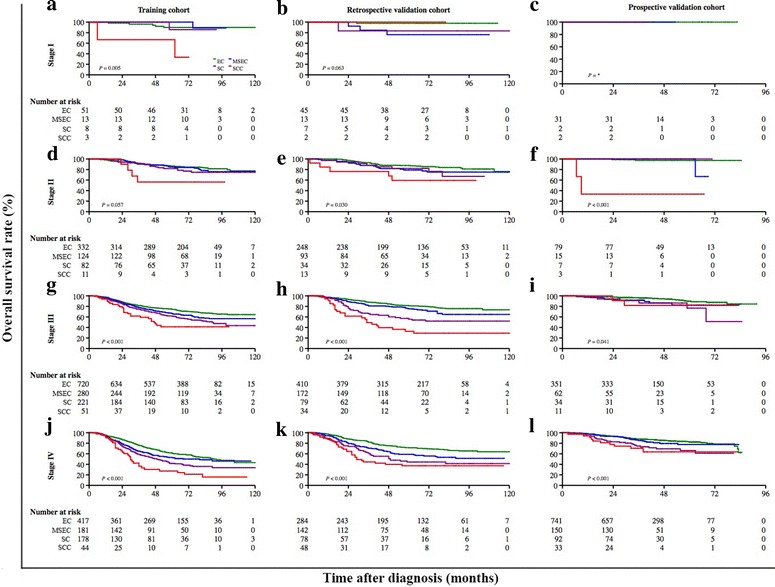
Kaplan-Meier survival estimates for NPC patients at different clinical stages in three separate cohorts stratified by subtypes according to the proposed classification. OS curves of NPC patients stratified by subtypes according to the proposed classification with stage I (*P* = 0.005, **a**; (*P* = 0.063, **b**; **c**), II (*P* = 0.057, **d**; *P* = 0.030, **e**; and *P* < 0.001, **f**), III (*P* < 0.001, **g** and **h**; *P* = 0.041, **i**), and IV disease (*P* < 0.001, **j**, **k**, and **l**) in the training, retrospective validation, and prospective validation cohorts, respectively. The log-rank test was used to calculate *P* values. *No test possible because there were no failures

**Fig. 8:**
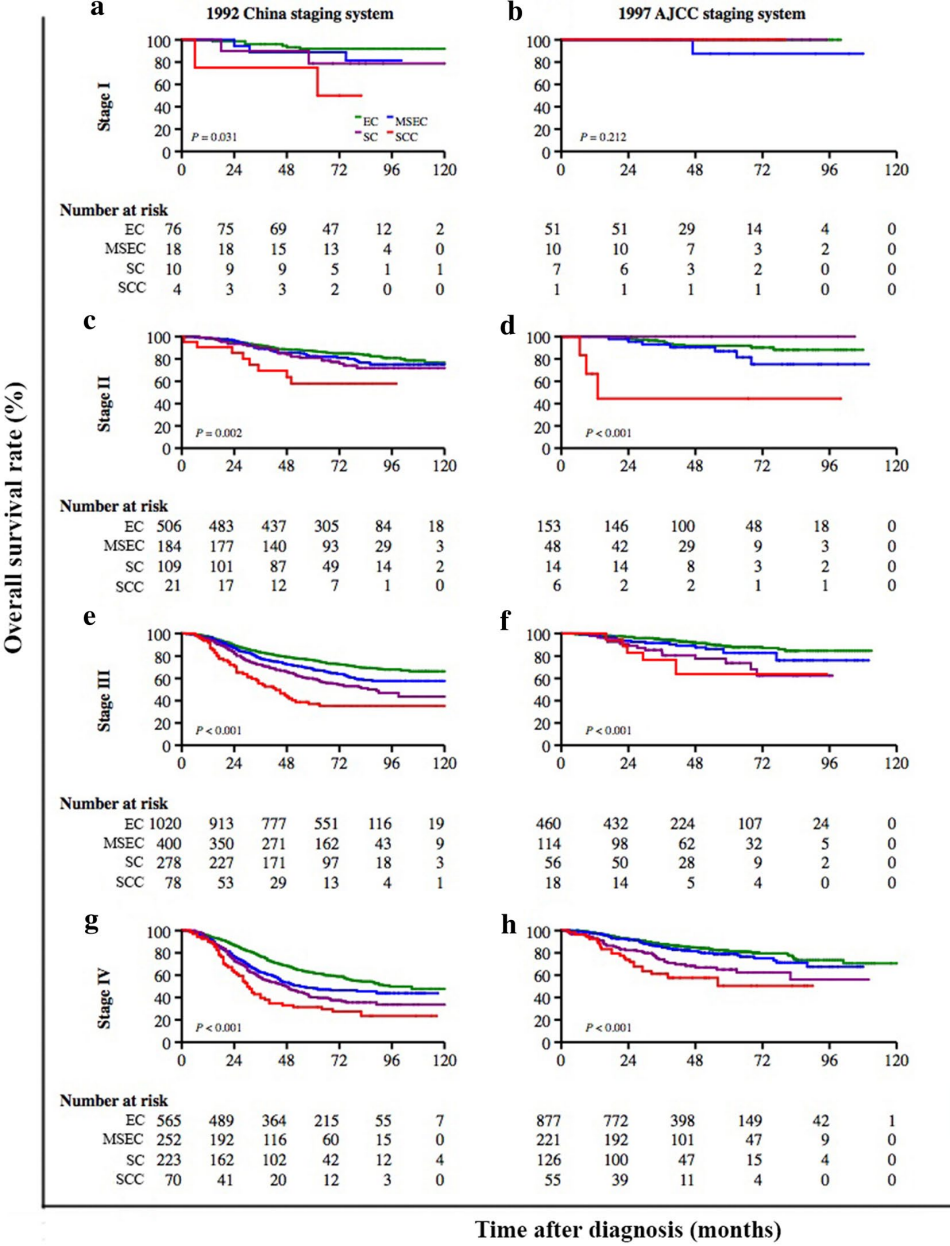
Kaplan-Meier survival estimates for NPC patients at different clinical stages according to two staging systems and stratified by subtypes according to the proposed classification. The OS differed significantly according to subtype of the proposed classification for patients at different clinical stages classified according to the 1992 China staging system (*P* = 0.031, **a**; *P* = 0.002, **c**; *P* < 0.001, **e** and **g**) and the 1997 AJCC staging system (all *P* < 0.001, **d**, **f**, and **h**), respectively. The log-rank test was used to calculate *P* values

Different clinical staging systems were adopted in this study. The 5-year OS rate was 69.2% (95% CI = 67.7%–70.6%) for patients who were classified according to the 1992 China staging system (Fig. 4a) and was 82.2% (95% CI = 80.3%–83.9%) for patients who were classified according to the 1997 AJCC staging system (Fig. 4b). The association between the proposed classification and clinical stage was statistically significant when both staging systems were combined (Chi square test, *P* < 0.001; Fig. 5). After stratification by the combination of both clinical staging systems, the proposed classification still remained a significant predictor of prognosis in all NPC patients (Fig. 6). In the three cohorts that were examined separately, the proposed classification retained significance in the prediction of prognosis, especially in NPC patients with stages III and IV cancers, who accounted for approximately 80% of the enrolled patients (Fig. 7). This remained true irrespective of whether the clinical stage was determined according to the 1992 China staging system or the 1997 AJCC staging system (Fig. 8).

### Multivariate analysis of OS according to the proposed classification

The proportional hazards assumption for each covariate was tested by graphical methods, and no significant violations were found. In the univariate analyses of OS for all NPC patients, the subtypes of MSEC, SC, and SCC, older age, male sex, RT alone, and advanced clinical stage were associated with significantly shorter OS (Table 4). In the multivariate analysis, the proposed classification independently predicted OS even after adjustments for age, sex, therapeutic modality, tumor stage, and WHO classification. Compared with EC, the HR was 1.44 (95% CI = 1.27–162, *P* < 0.001) for MSEC, 2.00 (95% CI = 1.76–2.28, *P* < 0.001) for SC, and 4.23 (95% CI = 3.34–5.38, *P* < 0.001) for SCC (Table 4). After multivariate adjustment, the SC subtype predicted a higher risk of death compared with the MSEC subtype (HR = 1.40, 95% CI = 1.21–1.61, *P* < 0.001); moreover, SCC was associated with a higher risk of death compared with SC (HR = 2.11, 95% CI = 1.63–2.75, *P* < 0.001) (data not shown).

### Therapeutic efficacy of RCT versus RT alone

**Fig. 9:**
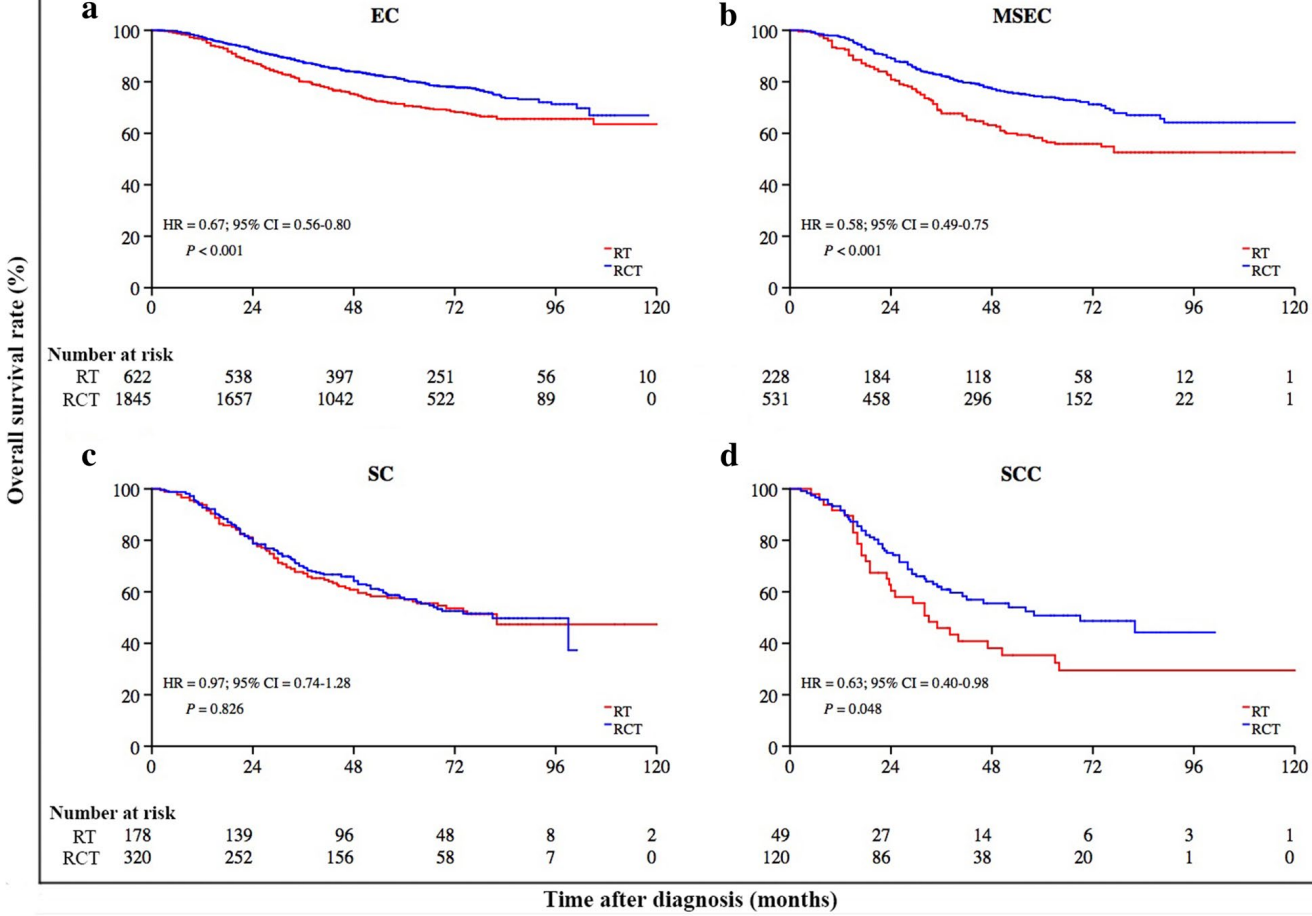
Kaplan-Meier survival estimates for the 3893 advanced NPC patients with different proposed subtypes who underwent radiochemotherapy (RCT) or radiotherapy (RT) alone. RCT significantly improved survival compared to RT alone for patients with EC (*P* < 0.001, **a**) and MSEC (*P* < 0.001, **b**), but not for patients with SC (*P* = 0.826, **c**); this was also likely the case for those with SCC (*P* = 0.048, **d**). The log-rank test was used to compute *P* values. Hazard ratios (HRs) with 95% confidence intervals (CIs) were computed for each type (RCT versus RT alone)

Overall, 3893 patients with advanced NPC who were diagnosed between 2001 and 2011 were stratified by the proposed classification and underwent further analysis to determine the therapeutic efficacy of RCT versus RT alone (Fig. 1). The baseline demographic and clinical characteristics of patients with advanced NPC who were treated with RCT (*n* = 2816) or RT alone (*n* = 1077) are shown in Table 5. The 5-year OS rates of patients with advanced NPC differed significantly between the RCT and RT alone groups (75.6% vs. 64.8%, *P* < 0.001). RCT prolonged OS as compared with RT alone for patients with the EC subtype (HR = 0.67, 95% CI = 0.56–0.80, *P* < 0.001; Fig. 9a) and the MSEC subtype (HR = 0.58, 95% CI = 0.49–0.75, *P* < 0.001; Fig. 9b), but not for patients with the SC subtype (HR = 0.97, 95% CI = 0.74–1.28, *P* = 0.826; Fig. 9c). However, this was also likely the case for those with the SCC subtype (HR = 0.63, 95% CI = 0.40–0.98, *P* = 0.048; Fig. 9d). A multivariate Cox analysis showed that after adjustment for age, sex, clinical stage, and the WHO classification, both therapeutic modality and the proposed classification were significant predictors of the survival of advanced NPC patients (Table 6).

**Table 5:** Clinical characteristics of 3893 patients with advanced NPC who were treated with different therapeutic modalities

Characteristic	RT alone	RCT	*P* value
Total (cases)	1077	2816	
Age (years)			<0.001^†^
Median (range)	48 (11–90)	47 (10–85)	
Follow-up time (months)			<0.001^†^
Median (range)	60 (2–120)	52 (2–120)	
Sex [cases (%)]			0.120*
Male	785 (72.9)	2120 (75.3)	
Female	292 (27.1)	696 (24.7)	
Clinical stage [cases (%)]			<0.001*
III	685 (63.6)	1144 (40.6)	
IV	392 (36.4)	1672 (59.4)	
Proposed classification [cases (%)]			<0.001*
EC	622 (57.8)	1845 (65.5)	
MSEC	228 (21.2)	531 (18.9)	
SC	178 (16.5)	320 (11.3)	
SCC	49 (4.5)	120 (4.3)	
WHO classification [cases (%)]			<0.001*
NKUC	889 (82.5)	2215 (78.6)	
NKDC	160 (14.9)	560 (20.0)	
KSCC	28 (2.6)	41 (1.4)	
OS rate (%)			<0.001^§^
5-year (95% CI)	64.8 (61.7–67.6)	75.6 (73.8–77.3)	

*NPC* nasopharyngeal carcinoma, *RT* radiotherapy, *RCT* radiochemotherapy, *EC* epithelial carcinoma, *MSEC* mixed sarcomatoid-epithelial carcinoma, *SC* sarcomatoid carcinoma, *SCC* squamous cell carcinoma, *WHO* World Health Organization, *NKUC* non-keratinizing undifferentiated carcinoma, *NKDC* non-keratinizing differentiated carcinoma, *KSCC* keratinizing squamous cell carcinoma, *OS* overall survival, *CI* confidence interval

* Chi square test

^†^Student’s *t* test

^§^Log-rank test

**Table 6:** Cox proportional regression analysis of associations between clinical characteristics and OS in 3893 patients with advanced NPC

Characteristic	OS rate (%)		Unadjusted HR	95% CI	*P* value	Adjusted HR^a^	95% CI	*P* value
	5-year	95% CI						
Therapeutic modality								
RT	64.8	61.7–67.6	1.00	Reference		1.00	Reference	
RCT	75.6	73.8–77.3	0.67	0.59–0.76	<0.001	0.64	0.56–0.72	<0.001
Proposed classification								
EC	78.3	76.5–80.0	1.00	Reference		1.00	Reference	
MSEC	69.1	65.4–72.5	1.48	1.27–1.72	<0.001	1.43	1.22–1.67	<0.001
SC	57.6	52.8–62.1	2.24	1.91–2.62	<0.001	2.13	1.81–2.51	<0.001
SCC	46.4	37.8–54.5	3.14	2.49–3.96	<0.001	3.99	2.97–5.35	<0.001
Age								
≤47 years	77.7	75.7–79.6	1.00	Reference		1.00	Reference	
>47 years	66.8	64.4–69.1	1.58	1.39–1.78	<0.001	1.51	1.34–1.71	<0.001
Sex								
Male	71.5	69.7–73.3	1.00	Reference		1.00	Reference	
Female	75.2	72.1–78.0	0.81	0.69–0.93	0.003	0.82	0.71–0.95	0.008
Clinical stage								
III	76.8	74.7–78.8	1.00	Reference		1.00	Reference	
IV	68.3	66.0–70.5	1.38	1.22–1.56	<0.001	1.48	1.31–1.68	<0.001
WHO classification								
NKUC	72.6	70.8–74.2	1.00	Reference		1.00	Reference	
NKDC	73.2	69.5–76.7	0.98	0.83–1.15	0.780	0.97	0.81–1.15	0.690
KSCC	60.9	47.7–71.9	1.66	1.13–2.41	0.009	0.47	0.29–0.76	0.002

*NPC* nasopharyngeal carcinoma, *OS* overall survival, *CI* confidence interval, *HR* hazard ratio, *RT* radiotherapy, *RCT* radiochemotherapy, *EC* epithelial carcinoma, *MSEC* mixed sarcomatoid-epithelial carcinoma, *SC* sarcomatoid carcinoma, *SCC* squamous cell carcinoma, *WHO* World Health Organization, *NKUC* non-keratinizing undifferentiated carcinoma, *NKDC* non-keratinizing differentiated carcinoma,* KSCC* keratinizing squamous cell carcinoma

^a^All models were adjusted for the proposed classification, age, sex, therapeutic modality, clinical stage, and WHO classification

Due to the unbalanced distribution of clinical stages III and IV between the RCT and RT alone groups (Table 5), we repeated this analysis after the included cases were restricted to either stage III or stage IV NPC. Consistent with the results for all advanced NPC patients, significant differences in the 5-year OS rates were observed between patients who were treated with RCT and RT alone within the subtypes of EC and MSEC after the cases were restricted to either stage III or stage IV cancers (data not shown).

## Discussion

Prognostic evaluation is pivotal for making decisions concerning appropriate treatment delivery. In our present study, we developed an NPC histopathologic classification that can distinguish among the different subtypes with clinically and statistically significant differences in the 5-year OS rate, even after multivariate adjustment for or stratification by TNM stage. Compared with the training and retrospective validation cohorts, the estimated 5-year OS rate was most likely higher in the prospective validation cohort due to the more widespread delivery of RCT to NPC patients seen between 2007 and 2011 at SYSUCC. An additional explanation for the higher 5-year OS rate in this cohort (83.5% vs. 68.7% and 73.3% in the other two cohorts) is that it also had the shortest median follow-up time (41 vs. 68 and 68 months in the other cohorts), which was due partly to the more recent diagnosis of these patients.

Considerable controversy surrounds the WHO classification and its prognostic value [22]. The WHO KSCC subtype accounts for one-third to one-half of all NPC cases in western populations, and this subtype is associated with a worse prognosis compared with non-keratinizing carcinoma [23, 24]. However, studies have consistently failed to show that the distinction between the WHO NKDC and NKUC subtypes has any clinical relevance [6–8]. These two subtypes comprise more than 95% of NPCs in endemic areas, including in our study population [4, 25].

RCT has consistently produced a survival benefit compared with RT alone, and 5-year OS rates of approximately 70% have been achieved by RCT in patients with stages III and IV NPC [12–15, 18, 26–30]. Lewis et al. [31] reported that 10.9% of patients received inadequate adjuvant therapy and 4.4% received inadequate radiotherapy based on the US National Comprehensive Cancer Network head-and-neck guidelines for recurrent or residual head-and-neck cancer. Exploratory analyses in our study indicated that compared with RT alone, RCT may improve outcomes in patients with advanced disease for the EC and MSEC subtypes, and possibly for the SCC subtype, but no evidence of an effect for the SC subtype was found. The worse prognosis of patients with the SC and SCC subtypes suggests that the current therapeutic methods are insufficient for these disease subtypes. Our results indicate that the proposed classification system may enable a more tailored approach for optimal clinical treatment decisions, especially for the SC and SCC subtypes, in which patients may need aggressive therapies such as high-dose irradiation, neoadjuvant and/or adjuvant chemotherapy, surgery, and molecularly targeted therapy to yield additional therapeutic gains. The efficacy of such therapies will require randomized clinical trials that stratify patients by histopathologic subtypes.

In the present study, the MRI and CT information of some patients are unavailable, particularly for the training cohort. The 1997 AJCC staging system is not applicable for these patients. Therefore, the 1992 China staging system was used. Our data clearly showed that different staging systems consistently predict the prognosis of NPC patients. Differences between these two staging systems mainly involve the classification of the borders of the tumor and lymph nodes in advanced disease [32]. Furthermore, Hong et al. [33] reported a high degree of similarity (72.1%) between the 1992 China staging system and the 5th edition of the AJCC staging system for NPC; the latter was then developed into the 6th AJCC system with minimal modification. In another study that compared the 5, 6, and 7th editions of the AJCC staging system in a total of 985 NPC patients, a minimal magnitude of improvement in prognostication was found [34]. Therefore, the 1992 China and 1997 AJCC staging systems were both acceptable for the prediction of prognosis.

A limitation to consider is in terms of generalizability because all included patients were from NPC-endemic areas in East Asia. The replication of our results in non-Asian patients needs to be confirmed for the use in clinics worldwide. We had incomplete follow-up data for 24% of the cohort, which raises the possibility of selection bias; however, differential follow-up based on histopathologic subtype was unlikely. The distribution of clinical stages differed between the RCT and RT alone groups, in which the patients were not randomized. Therefore, the efficacy of therapeutic strategies in patients with different histopathologic subtypes should be explored in future randomized clinical trials with complete follow-up. We cannot exclude the possibility that shorter survival in some groups is due to comorbidity, performance status, or some other unmeasured prognostic factors, rather than histopathologic subtype. However, no prior studies have demonstrated an association between histopathologic features and comorbidity or performance status, which indicates that they are unlikely to be strong confounders. Finally, complex gene networks may be the underlying mechanisms for the morphologic traits; thus, genomic analyses are warranted for further investigation and verification to elucidate the molecular basis for the proposed classification.

In conclusion, this multi-center study proposes an NPC histopathologic classification system that can significantly distinguish prognosis beyond clinical stage among non-squamous subtypes of NPC. The finding that RCT improves survival over RT alone in patients with advanced EC and MSEC suggests that more attention should be paid to the improvement of clinical outcomes for the SC and SCC subtypes, which are associated with a worse prognosis.
